# Talent identification research: a bibliometric study from multidisciplinary and global perspectives

**DOI:** 10.3389/fpsyg.2023.1141159

**Published:** 2023-05-25

**Authors:** Fabio Andres Parra-Martinez, Jonathan Wai

**Affiliations:** ^1^Wai Lab, Department of Education Reform, University of Arkansas, Fayetteville, AR, United States; ^2^Department of Psychology, University of Arkansas, Fayetteville, AR, United States

**Keywords:** talent identification, multidisciplinary research, sports, management and business, gifted education, bibliometrics

## Abstract

This paper describes the general status, trends, and evolution of research on talent identification across multiple fields globally over the last 80 years. Using Scopus and Web of Science databases, we explored patterns of productivity, collaboration, and knowledge structures in talent identification (TI) research. Bibliometric analysis of 2,502 documents revealed talent identification research is concentrated in the fields of management, business, and leadership (~37%), sports and sports science (~20%), and education, psychology, and STEM (~23%). Whereas research in management and sports science has occurred independently, research in psychology and education has created a bridge for the pollination of ideas across fields. Thematic evolution analysis indicates that TI has well developed motor and basic research themes focused on assessment, cognitive abilities, fitness, and youth characteristics. Motor themes in management and sports science bring attention to talent management beyond TI. Emerging research focuses on equity and diversity as well as innovation in identification and technology-based selection methods. Our paper contributes to the development of the body of TI research by (a) highlighting the role of TI across multiple disciplines, (b) determining the most impactful sources and authors in TI research, and (c) tracing the evolution of TI research which identifies gaps and future opportunities for exploring and developing TI research and its broader implications for other areas of research and society.

## 1. Introduction

Talent identification (TI) is paramount to the pursuit of excellence and human progress (Simonton, 2009; Makel et al., 2016). Conceived by some as a matter of national security and economic competitiveness, modern TI practice and research emerged in the US as a means to guarantee the country’s scientific prominence (Edgerton and Britt, 1943; Bush, 1945; Compton, 1951). Edgerton and Britt’s (1943) publication of “The Science Talent Search” documented one of the first TI deployments in K-12 settings focused on the Westinghouse Science Talent Search. Since then, TI has gradually become a concern of educators, policymakers, organizations, and researchers. TI embodies seeking and finding the most talented individuals who can solve real-world problems (Wai and Lovett, 2021), advance science (Lubinski and Benbow, 2006; Wai et al., 2009; Makel et al., 2016), enhance economic progress through innovation (Klett and Wang, 2013; Hsieh et al., 2019), and expand the limits of human performance and creativity (Till and Baker, 2020).

The research on TI exists primarily within the fields of gifted education (Olszewski-Kubilius, 1998; Lee et al., 2008; Subotnik et al., 2011; Assouline and Lupkowski-Shoplik, 2012; Boyanova, 2013; Matthews and Rhodes, 2020), sports sciences (Goncalves et al., 2012; Pankhurst and Collins, 2013) and management and business (Gallardo-Gallardo et al., 2015; De La Calle-Duran et al., 2021; Milani et al., 2021). TI research across sports and education focuses primarily on models, systems, programs, and processes to identify individuals with the potential for superior levels of academic or physical performance who can eventually make contributions to society (Faber et al., 2021). In management and business, TI is related to hiring practices to attract, screen, and retain high quality employees that warrant an organization’s competitiveness and leadership (Kotlyar, 2018; Johnson, 2020; Yogalakshmi and Supriya, 2020). Given the impact of TI for the advancement of human potential and capital, TI research has implications for individuals and their families, educators and coaches, and stakeholders and policymakers (McDonnell, 2011; McDonnell et al., 2020).

In the next section we briefly discuss definitions of talent, then provide context for our bibliometric approach to studying TI research.

### 1.1. Talent

Definitions of talent vary depending on the context and field. In gifted education, Gagne (Gagne, 1999) broadly defines talent as the result of transforming “high aptitudes into the well-trained and systematically developed skills characteristic of a particular field of human activity or performance” (p. 230). Aptitudes, often passed on from parents and nurtured and developed with environmental support, encompass a variety of physical, psychological, and social attributes that sustain the promise of individual success (Dohme et al., 2019; Faber et al., 2021). In a meta-analysis and systematic review of sports, Kusdinar and colleagues (Kusdinar et al., 2021) emphasized that TI focuses on detecting individuals who have the “potential to become elite players” if provided with a training environment that is very suitable for realizing potential in pursuit of excellence” (p. 1260). A complex concept, whether a measurable aptitude or a latent potential, many TI researchers have moved from the debate about the nature of talent toward the urgency of identifying and nurturing it. Ultimately, the goal of TI is to purposefully select talents leading to the building of talent pools (De La Calle-Duran et al., 2021; Jooss et al., 2021, 2022), promotion of elite performance (Allen and Hopkins, 2015), development of expertise (Ackerman, 2014; Hambrick et al., 2016; Dall Jensen et al., 2020; Dixson et al., 2020; Philip, 2022) and strategic use of talent for economic growth (Shen, 2017; Farndale et al., 2022). Hence, TI as well as subsequent talent development and allocation, have consequences for global innovation, technology, science, and economic growth (Hsieh et al., 2019).

Some characteristics of TI deployment and research are shared across fields. For example, TI researchers in education, sports, and psychology are concerned with early identification practices, adequate resources to nurture identified talent, and mechanisms to sustain high performance over time (Subotnik et al., 2011; Dixson et al., 2020; John and Thiel, 2022). From promise for elite athletic performance to prowess in science or outstanding work productivity in the professions, the fields of sports, education, and management have independently advanced TI research by determining what indicators of potential such as individual characteristics (MacNamara and Collins, 2015a; Gagné, 2018; Dohme et al., 2019) and talent-specific behaviors hint at potential for great performance (Schultz, 2018). Across fields, TI researchers seek to understand the mechanisms to detect and measure the extent of talented individuals’ characteristics and behaviors (Reilly et al., 2000; Vaeyens et al., 2008; Johnston et al., 2018). Moreover, TI relies on the predictive validity of assessments to ensure that present performance or characteristics are indeed antecedents to future success for the individual, organizations, and the betterment of society (Swiatek, 2007; Allen et al., 2015; Till et al., 2016; Johnston et al., 2018; Dobbin et al., 2019).

Determining who has potential or talent is not an easy task. TI requires decision-making and the involvement of stakeholders, making TI a dynamic, complex, and resource-intensive process (Abbott and Collins, 2004). The TI infrastructure consists of specialized personnel (e.g., educators, coaches, psychologists) equipped with sound multidimensional instruments to test, filter out, and select candidates for talent development programming or talent allocation, which naturally follows identification. Mobilizing resources to select a few recipients of talent development support is often high stakes, leading TI practices to face several challenges. Issues about reliability and validity are common challenges to the consistency and appropriateness of TI assessment tools, procedures, and selection methods (Faber et al., 2014; Larkin and O’Connor, 2017; Dobbin et al., 2019; Höner et al., 2021). Threats to validity in talent selection methods can have severe consequences for individuals and organizations (Jokuschies et al., 2017). Additionally, critics of TI have raised concerns about the clarity and accuracy of talent as a set of constructs. Arguing for a constructivist view of talent and TI, John and Thiel (2022) view talent as “a social construction that is historically changing and contextually embedded” (para. 16). Therefore, the TI process and selection of talents can be obscured by subjective judgments of stakeholders and lack of consensus. Challenges to the objectivity of TI have been linked to varying conceptions of talent and the measurable characteristics of talented people (Wiblen et al., 2012). Furthermore, equity and justice have received increased attention in TI research and practice in recent years (Brown and Wishney, 2017; Khoreva et al., 2019). Power dynamics and stakeholder-based interests raise questions about the reliability, transparency, and fairness of the identification and selection processes to ensure representation and diversity of traditionally underserved populations (MacNamara and Collins, 2015a; Larkin and O’Connor, 2017; McDonnell et al., 2020; Peterson et al., 2022; Bolckmans et al., 2022).

Considering the above-mentioned perspectives, we conceptualize TI as the process whereby stakeholders devise strategies to detect potential for high achievement in selected areas of human performance across the life span. This definition allows flexibility to incorporate perspectives on talent as characteristics, aptitudes, and potential for high performance in settings like academics (i.e., gifted education), cognitive abilities (i.e., psychology), physical abilities (i.e., sports and arts), as well as employee performance and productivity within organizations (i.e., management and business). We intentionally include the field(s) of management, business, and leadership that conceptualize talent as human capital in the workforce. This view allows us to highlight the implications of TI for lifelong personal development.

### 1.2. Bibliometric analysis

Bibliometric analysis is a strategy to map scientific work to identify the main streams of research over a period of time, contexts, and fields (Van Eck and Waltman, 2007). We take 1943–2022 as the time span for our analysis. The first article referenced in the Web of Science (WOS) and Scopus was the Edgerton and Britt (1943) paper which documented the Westinghouse Science Talent Search (now known as the Regeneron Science Talent Search) in 1943, a national competition that has been a major effort to identify scientific talent in high school seniors in the US since 1942 (Subotnik and Steiner, 1993). Bibliometric analysis also facilitates the understanding of the structure and evolution of a field given its patterns of productivity, influence, and collaboration around a research topic (Donthu et al., 2021). Therefore, 1943 is a reasonable starting point to examine the growth of TI research as well as its expansion across fields and international boundaries.

To date, a few bibliometric studies related to TI have been published. These studies have approached TI at the subdiscipline level: for example, Zheng and Liu (2022) examined research in physical education and identified the highest performing researchers within the field, by noting their number of citations and relative influence. Another study focused on a highly niche topic in sports science that investigated the relative age effect by which biases in talent selection exist due to the distribution of individual birth dates (Bilgiç and Işın, 2022). This prior work emphasized the role of timing and universal screening in TI as well as the effects of individuals’ age and readiness to receiving talent development support (Bilgiç and Işın, 2022). Another example within a restricted discipline focused on the integration of talent and labor management (Barkun et al., 2020). Barkun and colleagues addressed the use of the term talent within the labor market field, identifying the use of talent as a synonym of human capital rather than only an expression of high performance.

Our current paper aims to describe trends in TI research across multiple fields over the last 80 years to ultimately understand the past, present, and future of TI research. This bibliometric analysis provides new ways of viewing TI by implementing a performance and science mapping strategy. First, we use performance analysis to identify the scholarly productivity of researchers and the journals’ influence through publication and citation patterns. Second, we use science mapping to explore the collaborative, conceptual, and country-level networks in TI research. Bibliometric analysis also has the potential to motivate cross-disciplinary pollination of ideas (Donthu et al., 2021). Thus, in our discussion, we elaborate on the implications of cross-field trends by integrating suggestions for future directions in the study of TI.

### 1.3. Research questions

We aimed to answer the following research questions:

RQ1. What are the patterns of publications and citations on TI from 1943 to 2022?

The goal of this question is to determine the annual scientific production and growth estimated by volume and document citation from a multidisciplinary angle to TI since the publication of Edgerton and Britt (1943).

RQ2. Who are the leading and influential researchers in TI?

The goal of this question is to produce a list of the most cited and productive researchers in TI across research fields.

RQ3. What are the top journals in TI research?

The goal of this question is to explore publication venues in order to understand academic communities that study TI. This question has the potential to identify research areas that can benefit from cross-pollination of research ideas among TI fields.

RQ4. Which countries’ scholarly productions on TI are most frequently found in the Scopus-WOS databases between 1943 and 2022?

The goal of this question is to determine collaboration networks across countries and which countries are more visible or impactful in TI research.

RQ5. What is the intellectual structure of the knowledge base on TI?

The goal of this question is to identify the patterns of scientific collaboration among researchers by mapping co-authorship and pattern of co-citation of influential works across fields.

RQ6. How have thematic and conceptual trends in TI evolved?

The goal of this question is to identify the most frequent keywords used in TI, mapping networks of key concepts, and producing cluster analysis and time slicing maps of recurrent themes in the adoption of keywords over time.

## 2. Methods and materials

### 2.1. Search strategy and data preparation

We conducted the term search on Scopus and WOS Core Collection on *October 31, 2022*. Both databases broadly cover the areas of science, the social sciences, and humanities. We limited our search within title, abstract, and keywords (TITLE-ABS-KEY). We searched for the term “talent identification” and equivalent terms such as “talent search,” “talent detection,” and “talent management” as keyword terms cited in recent systematic reviews and meta-analyses of talent identification (Hodges et al., 2018; Faber et al., 2021; Kusdinar et al., 2021). These terms were concatenated with the boolean “OR” for title and simultaneously used in an abstract search with the boolean “AND.” We also included the term (AND develop*) as talent identification and talent development are often coupled terms, given that one common goal of TI is to provide the means for talent development of those individuals identified. The final search string for each of the databases was the following:

Scopus

[TITLE-ABS-KEY (“talent search” OR “talent identification” OR “talent management” OR “talent detection”) AND TITLE-ABS-KEY (develop*)] AND [LIMIT-TO (DOCTYPE, “AR”)]

WOS

[TI = (“talent search” OR “talent identification” OR “talent management” OR “talent detection”)] OR AB = [“talent search” OR “talent identification” OR “talent management” OR “talent detection”] AND [AB = (develop*)]

The search produced 4,555 document references: Scopus (*n* = 1,943) and WOS (*n* = 2,612). We followed the Preferred Reporting Items for Systematic Reviews and Meta-Analyses (PRISMA) to structure our data. Because of the broad, global, and multidisciplinary focus of this paper, we did not filter results by a specific knowledge field, language, or time span. However, we filtered the results to only include peer reviewed journal articles (AR), using the database advanced filters. This decision was made on the basis that journal articles adhere more closely to publication indexation standards in reference formatting than other publication sources.

**Figure 1:**
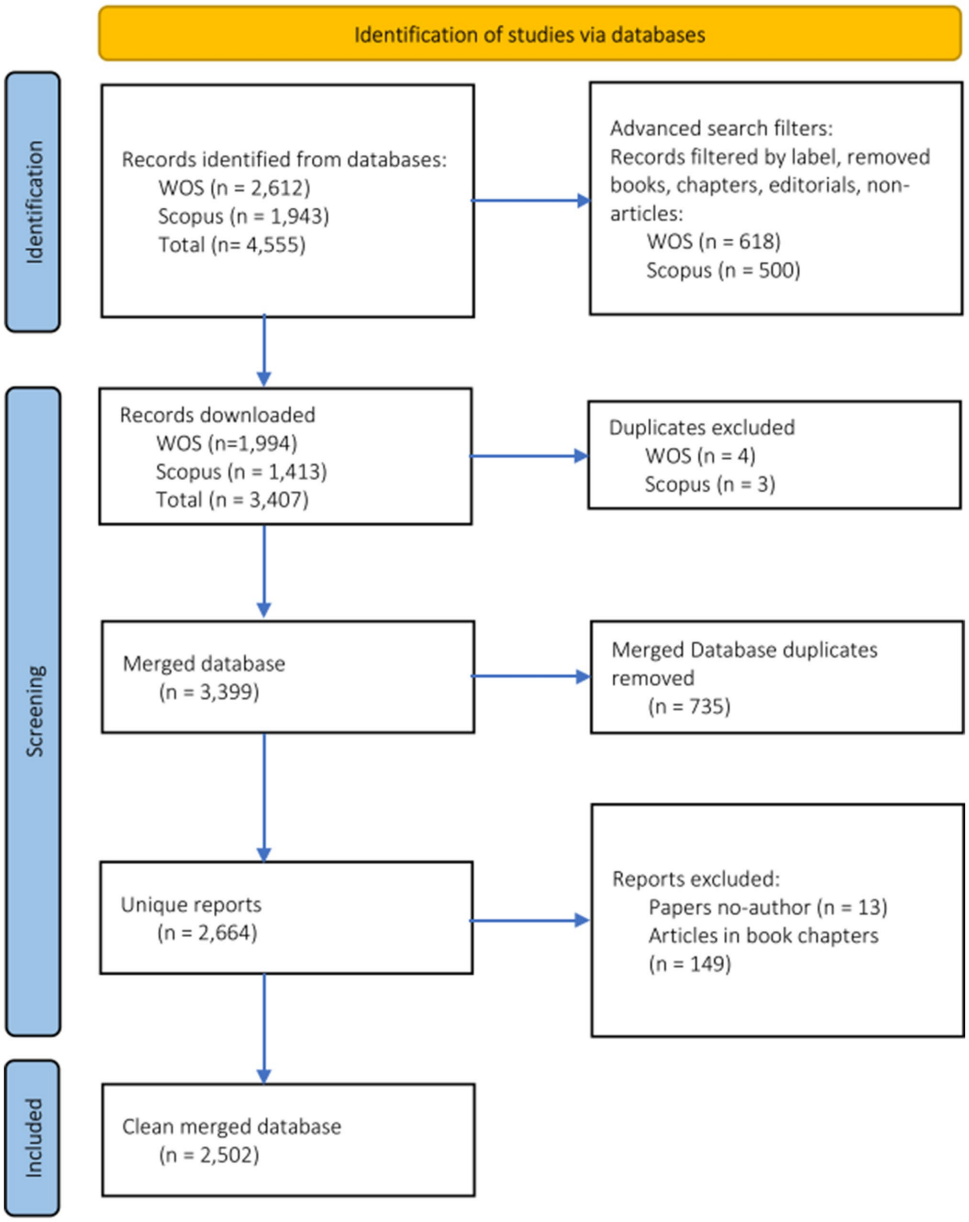
PRISMA flow diagram of the literature search strategy. PRISMA, Preferred Reporting Items for Systematic Reviews and Meta-analyses. Research studies about talent identification.

We downloaded the documents from Scopus and WOS in Bibtext format and merged the two databases using the R package “Bibliometrix” (Aria and Cuccurullo, 2017) and followed the recommendations that Echchakoui (2020) outlined in conducting bibliometric analysis combining data from Scopus and WOS. First, we converted the WOS and Scopus data to separate BIB files. Second, we replaced variable names with standard bibliometric metadata tags (e.g., author = AU, title = TI, references = CR, etc.). Separately, we cleaned the reference lists of each database using the code function MetaTagExtraction.^1^ Then, we merged the databases and transformed the resulting file to xlxs format. We eliminated 735 duplicate observations from the merged database using the metadata tag columns for article title “TI,” direct object identifier “DOI,” and Abstract “AB” as criteria for sorting and matching double observations. Then, we screened the resulting database for missing data observations (e.g., data lines with missing references, authors’ names, references, abstracts, etc.). We eliminated 13 data lines marked with author “anonymous,” or “NA” and 149 data lines that were classified as “Article: Book Chapter.” The latter were generally articles included in handbooks. The final dataset included 2,502 unique observations, 43 unique variables representing 5,380 authors, 920 publication sources, and 12 languages. Figure 1 shows the PRISMA flowchart of screening and inclusion/exclusion criteria applied to the search results.

Since bibliometric data do not include meta-tags about field or research discipline, we created an additional column “field” in the data and added a classification category to each observation. To classify each article, we matched journal title keywords to their classification in the Scimago Journal and Country Rank (Scimagojr) website.^2^ This website contains a database of indexed journals and their field classifications. We downloaded the database and used it to match the Scopus and WOS database. When a journal title was not found in Scimagojr, the researchers assigned the classification tag manually by looking at the journal classification on the journal homepage or the Web of Science. The major field data tags included education, management, multidisciplinary study, psychology, sports science, as well as science, technology, engineering, and mathematics (STEM). These tags are extensive and inclusive of multiple related subdisciplines within each field. For example, management encompassed publications related to management, leadership, business, economics, and human resources, among others.

### 2.2. Data analysis

We conducted performance and mapping network analysis of our data using the R Bibliometrix package and the visualization tools Biblioshiny 4.0 (Aria and Cuccurullo, 2017) and VOSViewer (van Eck and Waltman, 2014). The Bibliometrix tool allowed us to implement fine grained performance analysis and compute statistics at different levels (e.g., country, journal, author, document). The Biblioshiny graphic interface and VOSviewer allowed us to visualize bibliometric networks and concept maps enhancing readability and accessibility.

To address scholarly productivity, we used author, journal, and article level metrics to rank the most influential items in the database. Metrics included global and local performance indices commonly used to measure the relative impact of publications. Specifically, we used the local *h*-index, *m*-index, and *g*-index. The *m*-index is a measure of publication citations whereby the index is the result of *h* number of articles with *h* number of citations. For example, an *h*-index of 10 signals that 10 publications under the same category have been cited at least 10 times. The *m*-index is the result of dividing the *h*-index by the number of years a journal or author has been active. The *g*-index is the largest perfect square in a list of articles, authors, or journals ranked by number of citations in descending order “such that the top *g* articles received together at least *g*^2^ citations.” The global *h*-index was extracted from the ScimagoJR website which offers current rankings for most online scientific publications. This global index represents all types of publications, not only those related to TI.

To analyze knowledge structures, we used network analysis with a focus on intellectual influence (co-citation), patterns of collaboration among authors and countries (social networks), and thematic maps using keyword clusters (conceptual maps) (Echchakoui, 2020). We used the Walktrap algorithm as our preferred clustering strategy for community detection. We applied the normalization association strength in order to determine the relatedness between items in our data (Van Eck, 2011). This exploratory approach allowed us to determine potential networks without *a priori* constraints (Smith et al., 2020). This approach allowed us to make comparisons across fields, authors, and journals. By grouping bibliometric elements, we were able to identify patterns of relatedness among similar elements. Since this analysis covered multiple fields, network visualization was key to determine how connected the fields are, and what key elements act as bridges connecting different, authors, documents, journals, and fields.

## 3. Results

Overall, the merged database included a wide variety of empirical and conceptual articles in the six discipline categories. Most articles related to TI were found in the field of management (~37%), followed by sports science (~20%). Education, psychology, and STEM categories only accounted for a combined (~23%) of all publications included in the analysis. A summary of the merged database characteristics is presented in Table 1. Whereas there was diversity in publication language (e.g., Spanish, Chinese, Turkish, Portuguese, French, and Russian), the vast majority of articles (2,319, or 92.7%) were published in English.

**Table 1:** General information merged database.

Description	Results
*Core information about data*	
Timespan	1943–2022
Sources (journals)	920
Documents	2,502
Annual growth rate	7.19%
Document average age	6.27 years
Average citations per document	18.65
References	84,999
*Document contents*	
Keywords plus (most common words in indexed database)	3,078
Authors keywords (manually provided by authors)	5,136
*Authors*	
Total authors	5,380
Unique authors of single-authored documents	372
*Author collaboration*	
Single-authored documents	411
Co-authors per document	3.03
International co-authorships (percentage)	27.38%
*Field*	
Management, business, and leadership	927 (37.05%)
Sports science	512 (20.46%)
Multidisciplinary	486 (19.42%)
STEM	255 (10.19%)
Education	177 (7.07%)
Psychology	145 (5.79%)

### 3.1. Research growth in TI from 1943 to 2022

**Figure 2:**
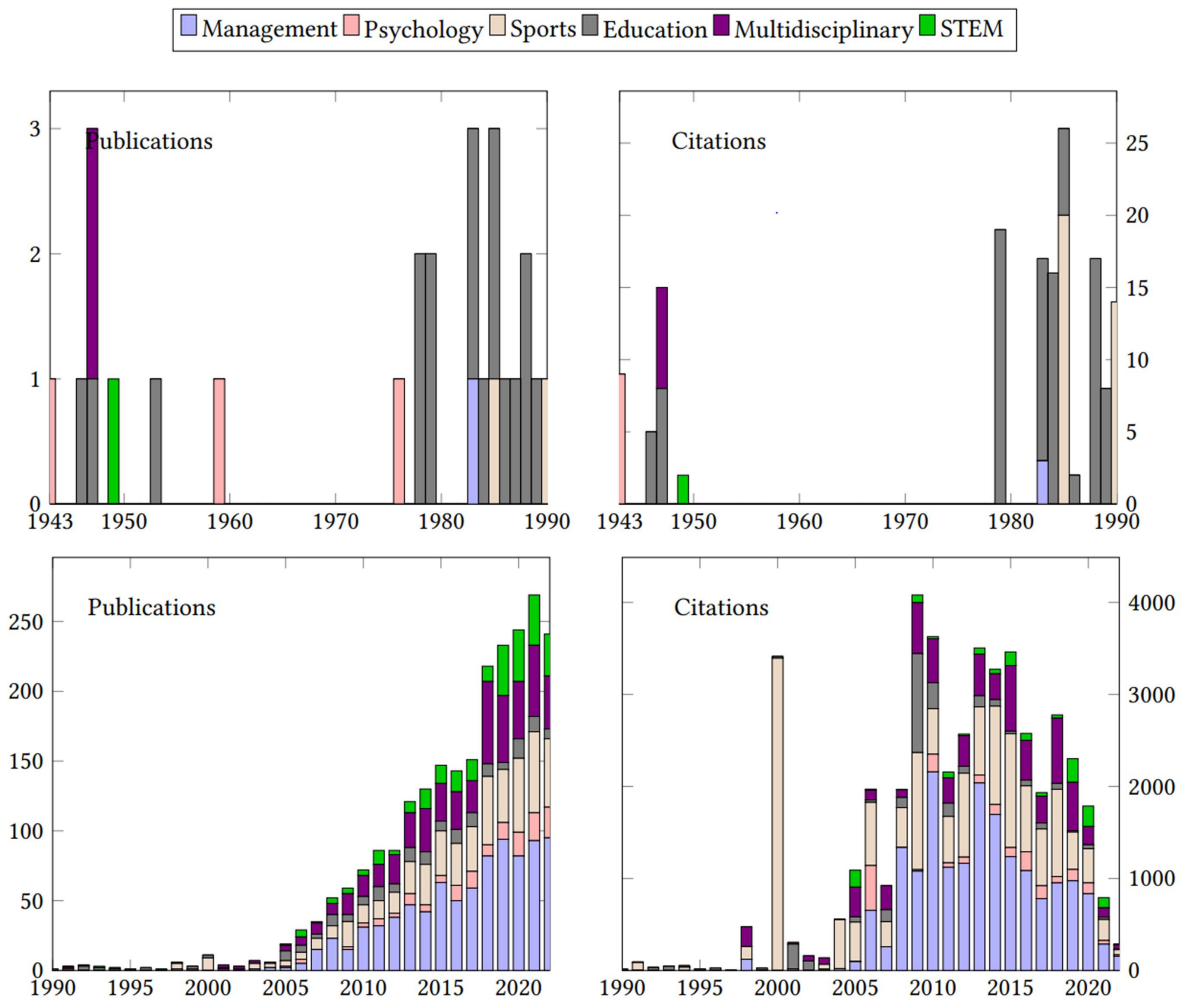
Total production and citation growth of TI research between 1943 and 2022.

The research in TI has maintained consistent growth over the past 80 years. The average year growth for research publication in TI across multiple areas was 7.19%. The number of publications and citations demonstrates the increasing interest in TI from multiple fields. Figure 2 presents the total production and citation growth in TI research including growth patterns disaggregated by research field according to the Scimagojr classification. The growth in TI research was slow during the second half of the 20^th^ century. In our data, only 62 (2.4%) articles were published between 1943 and 2000, corresponding to the fields of education, psychology, management, and sports. The fields of sports science and management showed an explosive growth pattern since the year 2000. All fields display a sharp upsurge of publications after 2000, which can be attributed to increased online publications and internet access. On average, the field with the most publications per year post 2000 was management (*M* = 37.87, SD = 33.9), followed by sports (*M* = 21.5, SD = 18.22) and multidisciplinary research (*M* = 20.4, SD = 17.6). Fields of education, psychology, and STEM produced, on average, fewer than ten articles per year according to Scopus and WOS databases. Two systematic reviews published in 2000 by Reilly and colleagues in the *Journal of Sport Sciences* caused the abrupt spike of citations in the field of sports.

### 3.2. Leading and influential researchers in TI

Table 2 shows the most productive and influential authors in TI according to Scopus and WOS index metrics. While the total number of publications is an indicator of productivity, we used the fractionalized publication count, which accounts for the number of co-authors in each publication. The total contribution of the top 30 out of 5,380 authors represents 9% of the total production of TI research and is concentrated in the fields of management and sports. An author’s impact is ranked by their *h*-index, a measure of impact based on the *h* number of articles with *h* number of citations. We ranked authors from multiple fields using this metric. Consistent with the accelerated growth in publications across fields, the top 30 researchers have continuously contributed to the literature of TI as revealed by their *h*-index, number of publications, and overall total citation count. Overall, the field of sports is well represented with 20 researchers, followed by management with seven researchers in the list. With the exception of Hugh Scullion and David G. Collings (in management), the top 10 most productive and influential researchers in TI published articles related to talent in sports. Only three researchers, David Lubinski and his collaborator Camilla Benbow (psychology and education) as well as Áine MacNamara (multidisciplinary) were classified within the most cited and influential researchers in TI. Although the *h*-index is regarded as a measure of academic influence, it is important to highlight that the *h*-index does not take into account whether an author publishes solo or with co-authors. Therefore, *h*-index alone is not an appropriate estimate for scientific productivity or author reputation (Koltun and Hafner, 2021).

**Table 2:** Top 30 researchers in TI ranked by *h*-index.

Rank	Author	*h*-index	*g*-index	*m*-index	Total citations	Total publications	Fractionalized publications	First publication	Main field
1	Collins D	17	21	0.81	1,182	21	8.5	2002	Sports
2	Vaeyens R	17	19	1	1,509	19	3.50	2006	Sports
3	Scullion H	15	18	1.154	1,292	18	6.45	2010	Management
4	Till K	15	24	0.652	596	24	5.26	2010	Sports
5	Lenoir M	14	21	0.875	707	21	4.20	2007	Sports
6	Philippaerts R	14	14	0.824	1,260	14	2.17	2006	Sports
7	Cobley S	13	18	1	530	18	3.67	2010	Sports
8	Williams A	13	16	0.52	2,555	16	5.37	1998	Sports
9	Fransen J	12	17	1.2	400	17	3.76	2013	Sports
10	Collings D	11	12	0.786	1,815	12	5.92	2009	Management
11	Malina R	11	11	0.611	1,289	11	1.86	2005	Sports
12	Baker J	10	19	0.667	443	19	4.80	2008	Sports
13	Macnamara A	10	13	0.667	489	13	3.17	2008	Multidisciplinary
14	Pion J	10	16	0.833	372	16	2.90	2011	Sports
15	Dries N	9	10	0.818	926	10	3.58	2012	Management
16	Robertson S	9	11	1.125	232	11	3.23	2015	Sports
17	Thunnissen M	9	9	0.9	787	9	4.33	2013	Management
18	Woods C	9	15	1.125	260	15	3.73	2015	Sports
19	Cooke C	8	8	0.615	336	8	1.78	2010	Sports
20	Cumming S	8	13	0.444	590	13	2.61	2005	Sports
21	Deprez D	8	9	0.8	294	9	1.40	2013	Sports
22	Hoener O	8	14	0.889	330	14	3.22	2014	Sports
23	Lubinski D	8	8	0.364	2,246	8	4.37	2001	Psychology/Education
24	Sparrow P	8	8	0.444	700	8	3.58	2005	Management
25	Vaiman V	8	11	0.667	529	11	4.00	2011	Management
26	Al Arris A	7	8	0.778	460	8	4.00	2014	Management
27	Benbow C	7	7	0.318	2,105	7	2.87	2001	Psychology/Education
28	Elferink-Gemser M	7	8	0.5	400	8	2.36	2009	Sports
29	Larkin P	7	10	0.583	202	10	3.20	2011	Sports
30	Bennett K	6	9	0.857	144	9	2016	1.92	Sports

*h*-index = *h* articles with *h* citations, *g*-index = top *g* articles with *g* citations, *m*-index = *h*-index divided by total number of active years. Total citations, number of publications, and year of first publication as found in the Scopus and WOS merged database.

### 3.3. Most relevant journals in TI research

We ranked the most relevant journals publishing TI articles using R-bibliometrix local *h*-index. This is the *h*-index calculated for all publications about TI included within the bibliometric database. As shown in Table 3, the TI publication *h*-index ranking of the top 30 sources produced a combined list of journals in the fields of management (*n* = 13), sports (*n* = 12), multidisciplinary (*n* = 3), psychology (*n* = 1), and education (*n* = 1). The most important journal in TI research is the *Journal of Sport Sciences* with a local *h*-index of 41, total of 114 publications in TI and over 7,400 citations. In contrast to the list of top authors, management-related publication venues dominate the list. The most prominent journal in management was the *International Journal of Human Resource Management* with a local *h*-index of 26, 73 publications, and 1,839 citations. Most high-ranked journal publications occurred within the last 10 to 20 years, which is consistent with the expansion of fields and the popularization of TI research. The average Scimago *h*-index for the top 30 journals was 90.1, which was greater than the average *h*-index of 72 for all journals included in the database. *PloS One* was the journal with the highest general *h*-index in the whole database. This multidisciplinary journal ranked seven with a local *h*-index of 14, and a general *h*-index of 367. Only two journals in psychology and education were included in the list. *Frontiers in Psychology* and *Gifted Child Quarterly* (GCQ) took positions 13 and 14, respectively.

**Table 3:** Most influential journals in TI research.

Rank	Journal	*h*-index	*g*-index	*m*-index	Total TI citations	Total TI publications	First TI publication	Scimagojr *h*-index	Field
1	Journal of Sports Sciences	41	85	1.413	7,481	114	1994	145	Sports
2	International Journal of Human Resource Management	26	41	1.444	1,839	73	2005	123	Management
3	Journal of World Business	24	24	1.846	2,893	24	2010	121	Management
4	Journal of Strength and Conditioning Research	23	40	1.211	1,641	47	2004	140	Sports
5	Human Resource Management Review	17	24	1	2,780	24	2006	101	Management
6	Journal of Science and Medicine in Sport	16	25	0.8	853	25	2003	108	Sports
7	Plos One	14	25	1.167	639	27	2011	367	Multidisciplinary
8	International Journal of Contemporary Hospitality Management	13	27	0.867	777	30	2008	100	Management
9	Human Resource Management	12	18	0.667	702	18	2005	100	Multidisciplinary
10	International Journal of Sports Physiology And Performance	12	19	0.8	391	23	2008	76	Sports
11	International Journal of Sports Science and Coaching	12	18	1	404	35	2011	31	Multidisciplinary
12	European Journal of Sport Science	11	21	0.687	481	23	2007	65	Sports
13	Frontiers in Psychology	11	18	1.222	381	38	2014	133	Psychology
14	Gifted Child Quarterly	11	18	0.244	364	23	1978	54	Education
15	Journal of Human Kinetics	11	19	0.786	392	23	2009	44	Sports
16	Employee Relations	10	24	1.428	602	29	2016	57	Management
17	European Journal of International Management	10	16	0.833	398	16	2011	28	Management
18	Human Resource Development International	10	14	0.714	334	14	2009	53	Management
19	Industrial and Commercial Training	10	17	0.667	383	17	2008	37	Management
20	Journal of Sports Medicine and Physical Fitness	10	16	0.5	263	16	2003	68	Sports
21	International Journal of Sports Medicine	9	11	0.643	743	11	2009	112	Sports
22	Biology of Sport	8	11	0.444	275	11	2005	33	Sports
23	Harvard Business Review	8	13	0.421	457	13	2004	190	Management
24	Personnel Review	8	14	0.615	291	14	2010	77	Management
25	Scandinavian Journal of Medicine and Science in Sports	8	10	0.667	342	10	2011	123	Sports
26	Science and Medicine in Football	8	14	1.333	219	25	2017	18	Sports
27	Thunderbird International Business Review	8	12	0.727	160	19	2012	42	Management
28	European Journal of Training and Development	7	12	0.636	237	12	2012	60	Management
29	International Journal of Performance Analysis in Sport	7	10	0.538	122	10	2010	34	Sports
30	Journal of Management Development	7	15	0.438	245	15	2007	63	Management

*h*-index = *h* articles with *h* citations, *g*-index = top *g* articles with *g* citations, *m*-index = *h*-index divided by total number of active years. Total citations, number of publications, and year of first TI publication as found in the Scopus and WOS merged database. Scimagojr *h*-index = total *h*-index for all journal publications.

**Figure 3:**
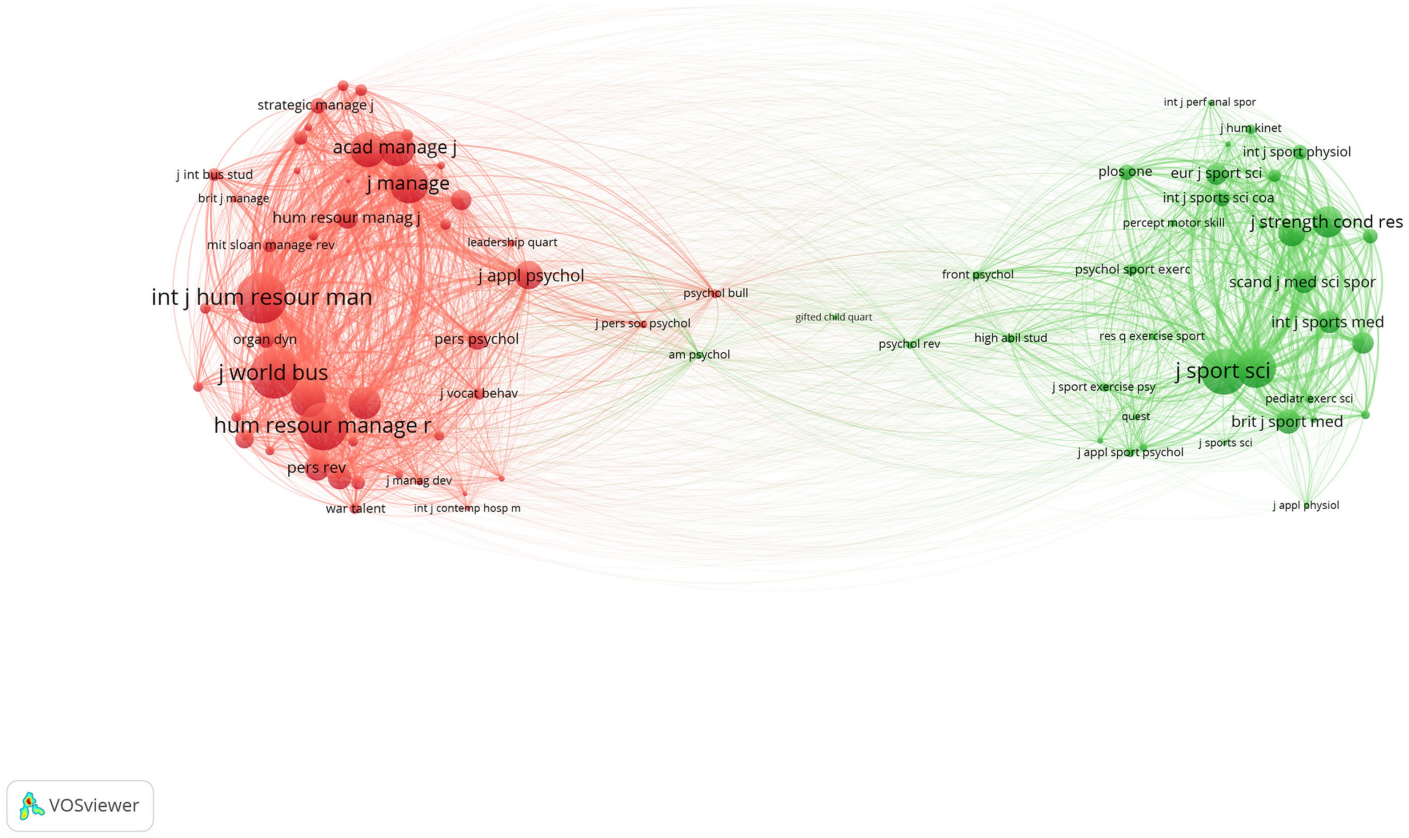
Bibliometric co-citation network for the Top 50 journals publishing TI research. Red = management cluster; Green = sports sciences. Journal abbreviations are in VOSviewer default style and size of text indicates relative prominence in the network.

Another important indicator of source relevance and influence is the pattern of co-citations across different journals. We produced the cluster and network represented in Figure 3. Data clustered in two main groups of journals. The field of management represented the largest cluster (in red), including journals related to business, human resource management, and administration science. The second major cluster (green) included journals related to sports sciences. When there is co-citation between two journals, a link is created between the nodes. Node proximity indicates how related the journals are, whereas frequency of co-citation is marked by the link strength or thickness. The size of the node is relative to the number of links with other journals and the frequency of co-citations. For example, the *International Journal of Human Resource Management* has the largest number of co-citations in the data followed by the *Journal of World Business* and *Human Resource Management Review*. These journals share the most links with other journals and a large number of co-citations with each other. The *Journal of Sports Sciences* has a strong connection with the *Journal of Sports Medicine* with many co-citations between the two journals. Of note, there is a bridge between the management and sports clusters. The bridge is comprised primarily by journals in psychology, education, and multidisciplinary research venues. Whereas education and psychology were underrepresented in this network, journals like *Frontiers in Psychology*, *Gifted Child Quarterly*, *High Ability Studies*, *Psychological Bulletin*, and *PloS One* serve as scholarly bridges for TI research across the most productive fields.

### 3.4. Country-level scholarly production and collaboration

Country-level scholarly production is largely influenced by a country’s scientific policy, resources, and access to research databases. As expected, out of 88 countries represented in the Scopus WOS data, the top ten countries were among the most industrialized economies and contributed 54.1% of the total publications in TI research. These countries include the United Kingdom (13%) and the United States (11.9%), as well as fast-growing economies like India (4.2%), South Africa (3.4%) and China (3.2%). By examining the volume of citations attained by country, the UK, the US, and Australia are the most influential countries with ~27% of the total citations in Scopus and WOS. Table 4 shows the summary of country-level production ranked by scholarly production. Based on the corresponding first author affiliation, we identified the percentages of collaborations among these most productive countries. Belgium was the most collaborative country with 48% of publications co-authored by at least one researcher from another country.

**Table 4:** Country-level production and collaborations.

Country	Total articles	Percent of total TI research	Single country articles	Cross-country articles	Percent collaboration	Total citations	Average citation per article
United Kingdom	326	13%	224	102	31.3%	10,806	33.15
United States	298	11.9%	242	56	18.8%	8,514	28.57
Australia	169	6.8%	97	72	42.6%	3,906	23.11
Spain	110	4.4%	84	26	23.6%	1,516	13.78
India	104	4.2%	92	12	11.5%	893	8.59
South Africa	84	3.4%	74	10	11.9%	492	5.86
China	81	3.2%	51	30	37%	547	6.75
Germany	80	3.2%	56	24	30%	1,554	19.43
Belgium	50	2%	26	24	48%	2,510	50.20
Canada	49	2%	32	17	34.7%	846	17.27

Country-level metrics for articles in the Scopus and WOS merged database.

**Figure 4:**
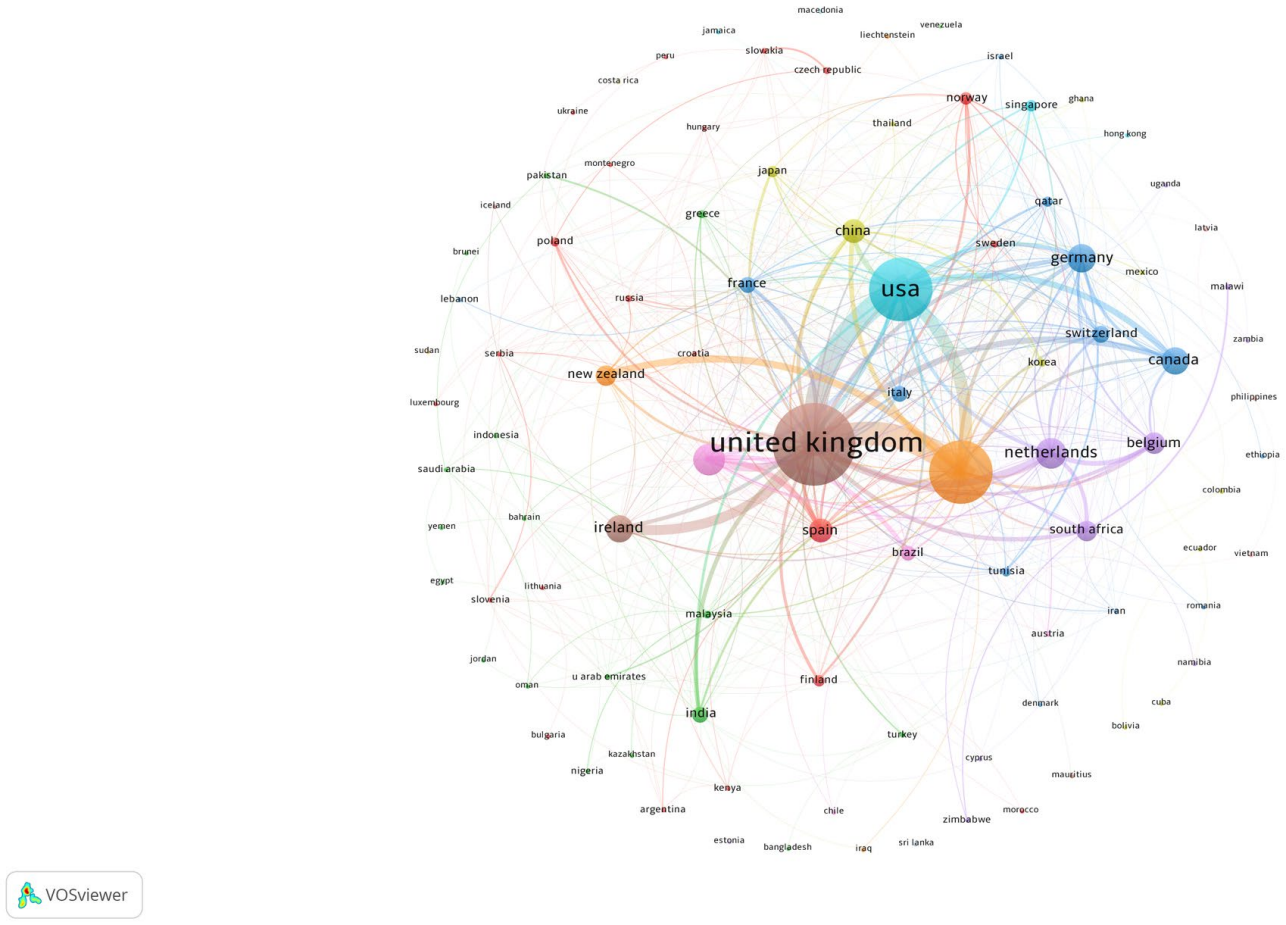
Cross-country collaboration network. Node size reflects total number of publications. Links between nodes represent frequency of collaboration.

Citation volume is a positive measure of a country’s influence on TI research. However, co-authorship analysis provides a stronger metric of a country’s influence. A closer look at cross-country dynamics reveals multiple collaborative clusters among countries (Figure 4). The UK had the largest collaboration network with 102 articles representing authors from 52 different countries. The US had a collaborative network with 45 other countries, totaling 56 articles. To maximize the visualization of all countries in the Scopus and WOS data, we set link strength at 1 co-authorship per document in the collaboration network. Countries with the most publications and collaborations are centered in the middle of the network, while countries with fewer collaborations and influence are located in the periphery. Eight countries only had one collaboration with another country (Bangladesh, Bulgaria, Bolivia, Brunei, Cuba, Jamaica, Mauritius, and Peru). Six countries did not have collaborations with other nations (Costa Rica, Estonia, Macedonia, Sri Lanka, Sudan, and Venezuela).

### 3.5. Intellectual structure of knowledge in TI research

**Figure 5:**
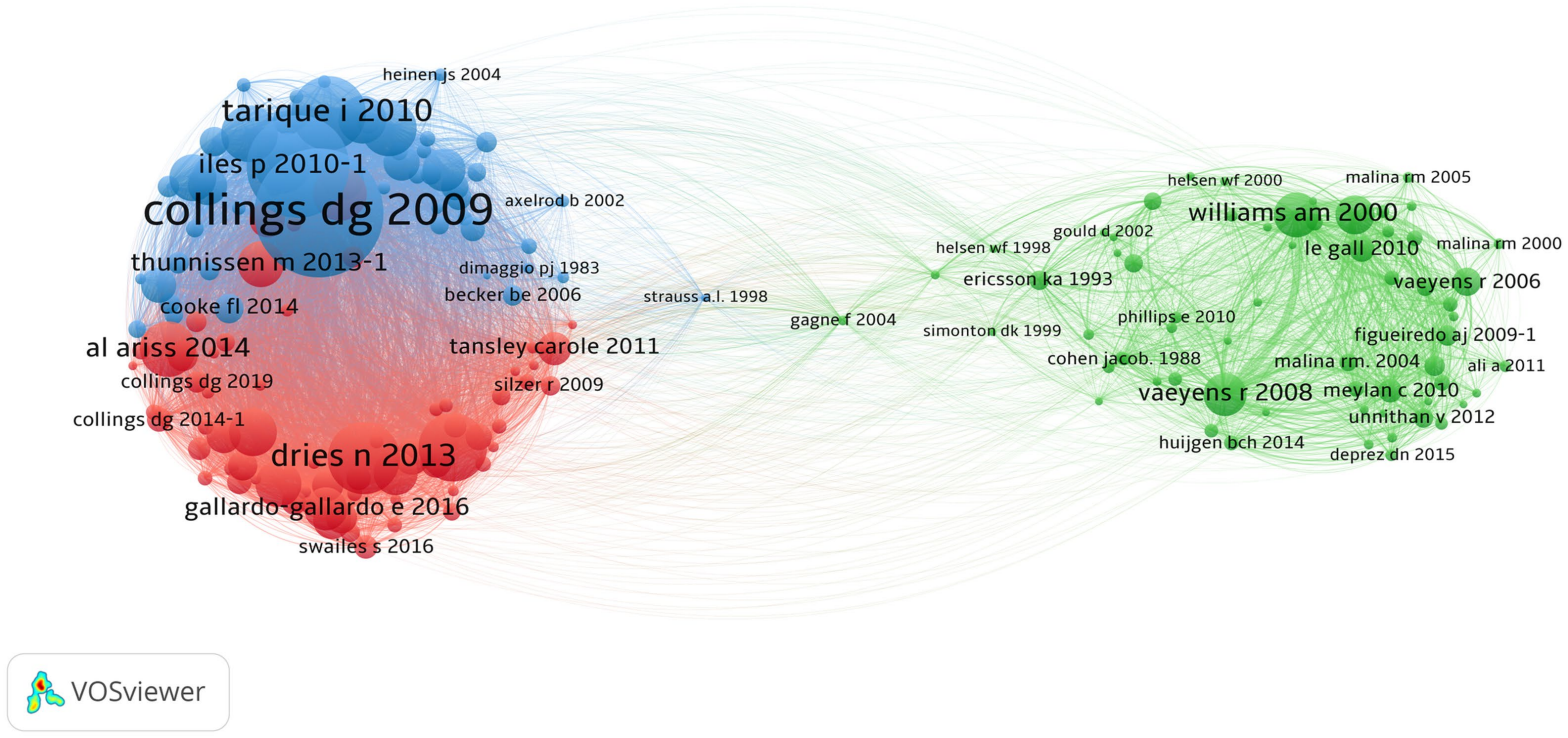
Co-citation patterns for most relevant articles in TI research. Author names are represented in the format last name and first and/or second initial. For example, collings dg 2009–1 indicates publication number 1 in the WOS and Scopus database led by David G. Collings published in 2009. Size of text and node represents relative level of prominence in the network.

We mapped co-citation networks across the most influential articles. Co-citation patterns indicate the number of times two different articles are concurrently cited in a new publication. Patterns of co-citation reflect the impact of research within a field. Figure 5 shows three clusters comprising 185 articles. Article level bibliometric coupling resembled the pattern of the scientific journal co-citation network, indicating potential strong research silos within management and sports science as well as the potential role of psychology and education researchers in connecting these two separate fields. Two foundational articles in gifted education, Gagne’s *Transforming gifts into talents* (Gagne, 2004), and the discipline of expertise, Ericsson’s *Role of deliberate practice on expertise* (Ericsson et al., 1993), bridged the TI research literatures of management and sports. The two largest co-citation clouds occurred in the field of management. The red cluster, with 66 articles, revolved around Al-Ariss et al.’s (Al Ariss et al., 2014) work reviewing theories of talent management and Gallardo-Gallardo et al.’s (Gallardo-Gallardo et al., 2013) paper questioning the meaning of talent in the workplace that is considered a foundational source in talent management. The blue cluster included 55 articles from which Collings and Melahi’s (Collings and Mellahi, 2009) review of strategic talent management had the most co-citations. The green cluster included 64 articles revolving around Reilly et al. (2000) and Vaeyens et al. (Vaeyens et al., 2008). These two articles were systematic reviews of articles focused on talent definitions, identification, and programming in sports. In particular, Reilly and Williams’ article is the most cited in our data (1,073 citations), playing a highly influential role in the study of perceptual skills as a component of soccer players’ talents.

**Figure 6:**
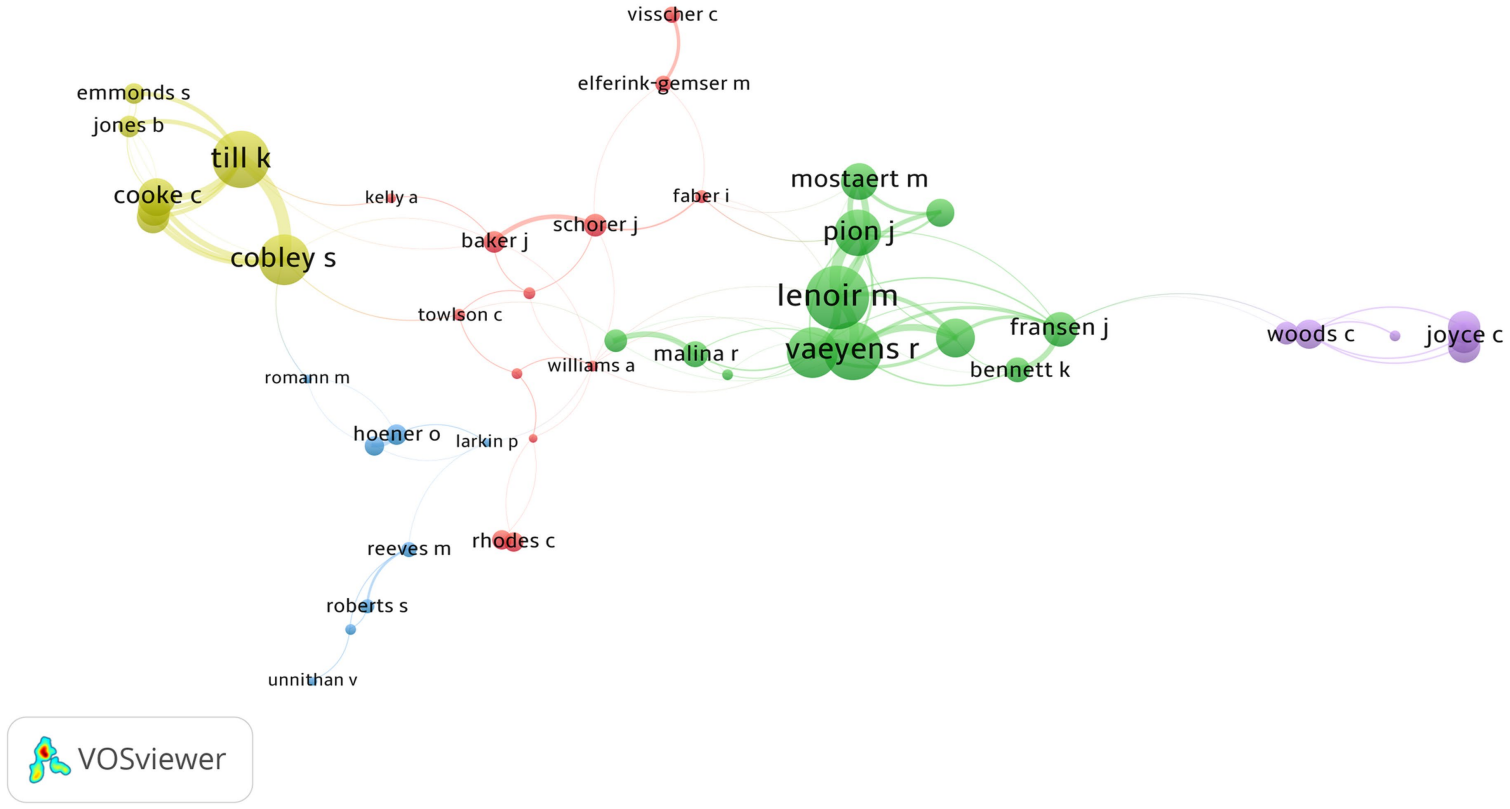
Collaboration network among researchers in Scopus and WOS. The strength (thickness) of a link indicates the frequency of collaboration between two authors. The size of a node and corresponding text size is relative to the number of collaborations.

Collaboration patterns are crucial to a field’s knowledge structure. The number of collaborations indicates support and concurrent interest in a stream or research theme. We determined collaboration patterns by mapping co-authorship in VOSviewer. Links were established for each collaboration between researchers based on a fixed number of five collaborations and their extended networks. Figure 6 shows a collaboration network with seven clusters. Dominant collaborations originated in the field of sports. The main cluster with 13 collaborators included prominent researchers such as Baker J, Schorer J, Kelly A, and others addressing challenges and solutions for optimal talent identification procedures in sports. Cluster two included 12 researchers such as Lenoir M, Vaeyens R, and Deprez D, who are leading collaborators in the area of anthropometric measures and task constraints as a means for talent detection and identification. Cluster three included Larkin P, Hoener O, and Unnithan V, who investigate identification instruments’ psychometric properties and validity. Cluster four was formed by researchers like Till K, Cobley S, and Cooke C who have concurrent collaborations on the topic of maturation and relative age in longitudinal evaluation of sports performance. The last cluster included Robertson S, Woods C, and Cripps AJ who are focused on issues of validity and reliability, multidimensional examinations, and coach bias. There were no clusters related to fields outside of sports science, which indicated that no single author in other fields had a network of at least four additional collaborators or collaborative publications with recurrent themes.

### 3.6. Thematic and conceptual evolution

Keyword frequency is a measure of the main themes of interest in a research field. We identified five main clusters grouping the top 250 keywords in TI research used since 1943. Table 5 shows the main keyword clusters in TI research.

**Table 5:** Author’s keywords and clusters.

Cluster 1	Count	Cluster 2	Count	Cluster 3	Count	Cluster 4	Count	Cluster 5	Count
Talent management	679	Talent identification	277	Talent	257	Somatotype	16	Talent search	12
Management	84	Soccer	70	Talent development	150				
Human resource management	73	Football	65	Performance	113				
Leadership	52	Anthropometry	62	Training	41				
Global talent management	49	Maturation	54	Motivation	29				
Human capital	46	Youth	39	Recruitment	26				
Talent retention	42	Expertise	38	Skills	26				
Succession planning	33	Adolescence	28	Coaching	24				
Human resources	31	Relative age effect	27	Gender	24				
Leadership development	31	Athlete development	25	Assessment	20				

**Figure 7:**
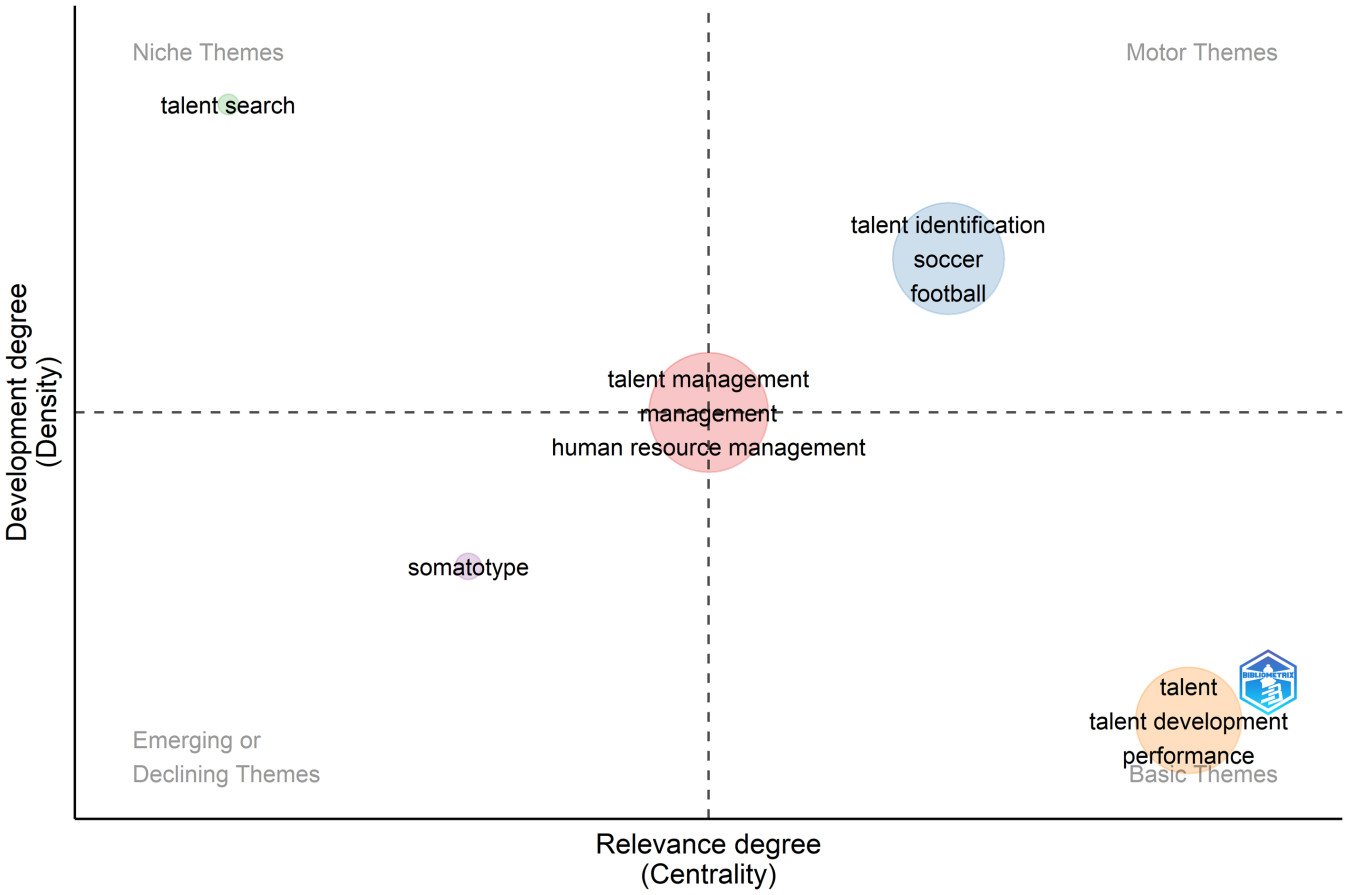
Thematic map of author’s keywords.

Keyword clusters provide insights into the extent of cohesion and development of a theme within a field. Therefore to further understand the thematic structure of TI research we produced thematic maps of authors’ keywords (Figure 7). A thematic map produces four quadrants measuring the level of importance or centrality of a research theme (number of relationships between themes) and the extent of development of a topic through measures of density (number of documents sharing the same keyword) (Cobo et al., 2011; Janik et al., 2021). The upper-right quadrant includes the field’s motor themes which are important and well-developed. The lower-right quadrant includes basic themes that are important but not well-developed. The upper left quadrant has established niches of research. Finally the lower-left quadrant has themes of low importance and low development these are either emerging or declining themes in the field (Chen et al., 2019)

**Figure 8:**
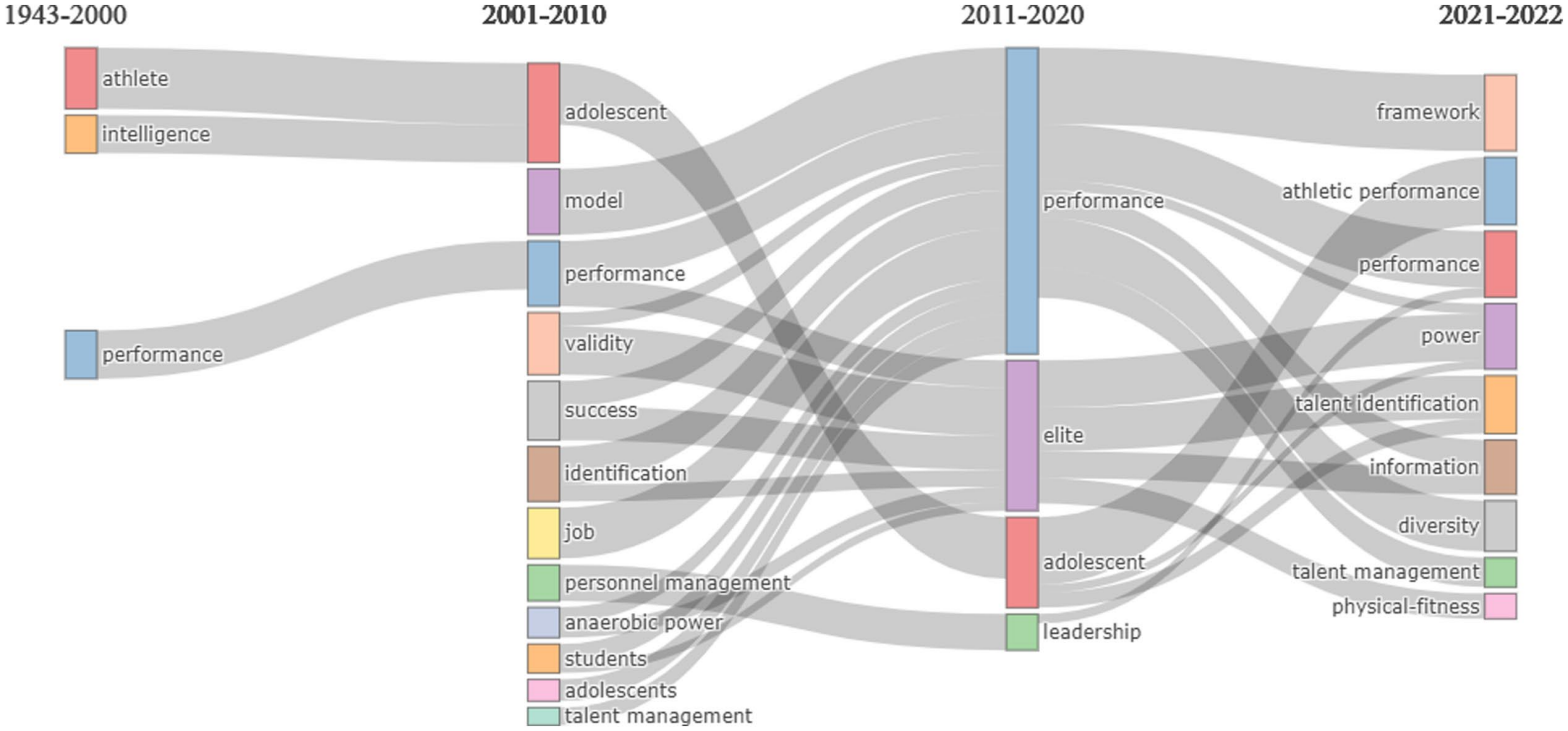
Evolution in TI research themes. Sankey plot presents the frequency of words used during each periods, while streams indicate the pattern of citation for emerging keywords.

Talent management was the most common keyword in the database (frequency: 679) forming the red cluster. This cluster was located at the intersection of the four quadrants indicating level of importance and development within the structure of knowledge in TI. Talent management keywords are related to the study of talent in the workplace. This cluster included words such as human capital retention, human resource management, leadership development, among others. The second most frequent word was “talent identification” forming the blue cluster (frequency: 277). This cluster was a motor theme indicating the increasing popularity of high level of development of TI in sports. This cluster included well-researched keywords such as anthropometry, football and soccer expertise, maturation and relative age, among others. The third most common word was “talent” forming the orange cluster in the basic quadrant (frequency: 257). Keywords like talent development performance training motivation indicate themes of high importance yet limited development in TI. The fourth cluster was formed by the word “somatotype” present in 16 studies studying the relationship of morphological profiles and performance. This theme had a relative low development and low importance which implies a declining tendency in TI research. Finally “talent search” by itself formed a small niched theme indicating high levels of development but limited in scope. Talent search in psychology and education is a specialized strategy for talent identification based on above-level testing in traditional academic contexts to provide sufficient headroom on the measurement tool to capture intellectual or academic performance at an early age (Assouline and Lupkowski-Shoplik, 2012). In other fields such as sports and management talent search is used as a synonym for talent identification or employee recruitment, respectively, Thematic evolution is concerned with the change in authors’ keywords over time. We produced time slices for keyword cluster analysis across three periods of time to examine changes in emerging, niche, basic, and motor research themes in TI. Our cutting points were (a) 1943–2000, marking the origins of TI research and slow initial growth during the first 50 years of TI research; (b) 2001–2010 as a period of increased activity in TI research and an explosion of thematic diversity, especially within sports science and management; (c) 2011–2020, a period consolidating the accumulated wealth of research in TI; and (d) 2021–2022, to connect current trends and emerging areas of research in TI. Figure 8 shows main research themes since Edgerton and Britt’s (1943) publication on TI.

**Figure 9:**
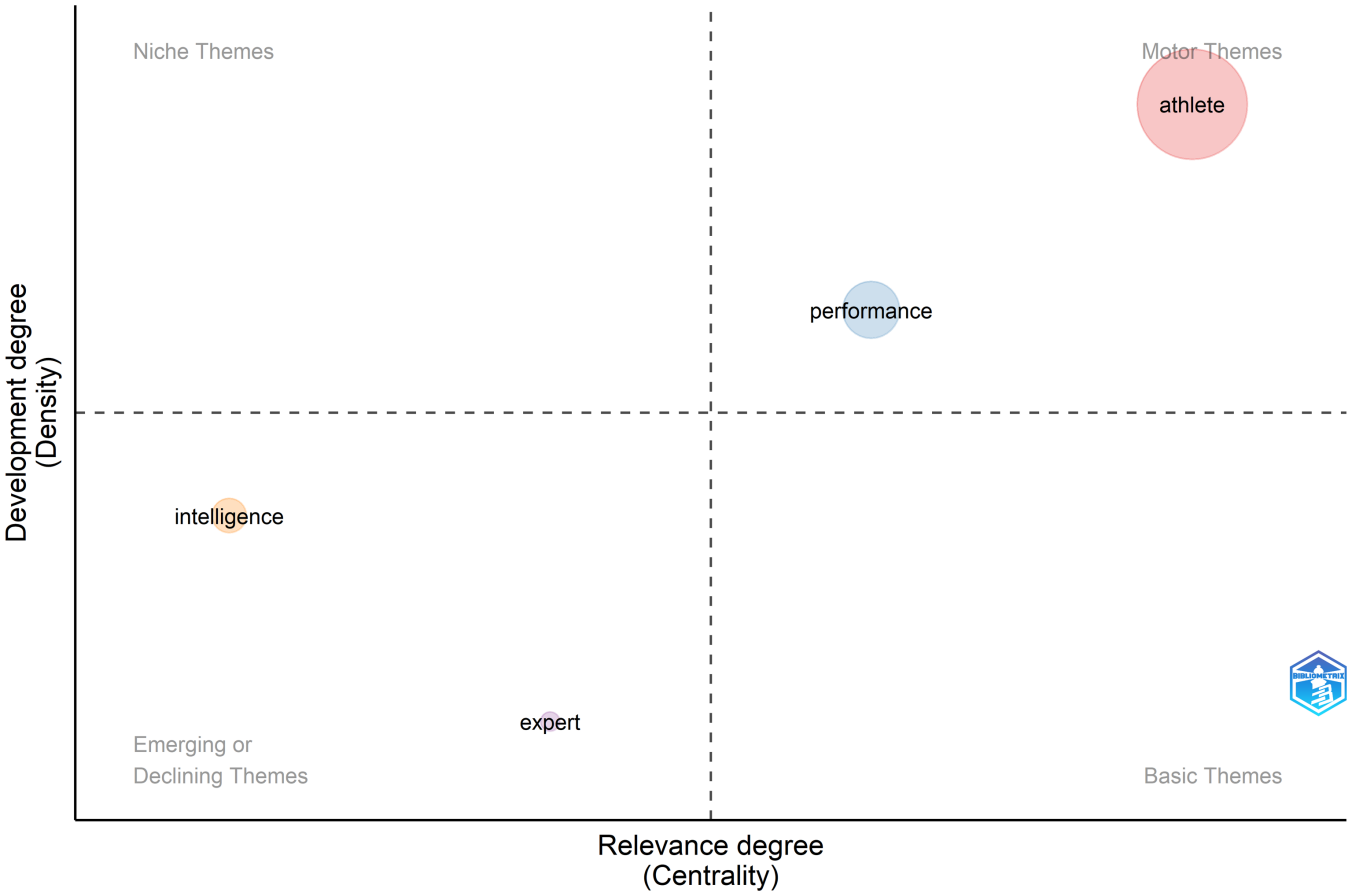
Thematic evolution from 1943 to 2000.

The first 50 years of TI research were characterized by few studies. Only 62 (2.4%) articles were published between 1943 and 2000 (Figure 9). During this period, two motor themes were related to the study of performance and physical characteristics of young athletes within the field of sports. Keywords related to athletes’ attributes (e.g., physiology, age factors, physical parameters), and performance (e.g., sports, skills, motivation) were the main keywords in TI. Emerging themes, largely occurring within the fields of education and psychology, focused on the study of intelligence (e.g., sex-differences), and development of expertise.

**Figure 10:**
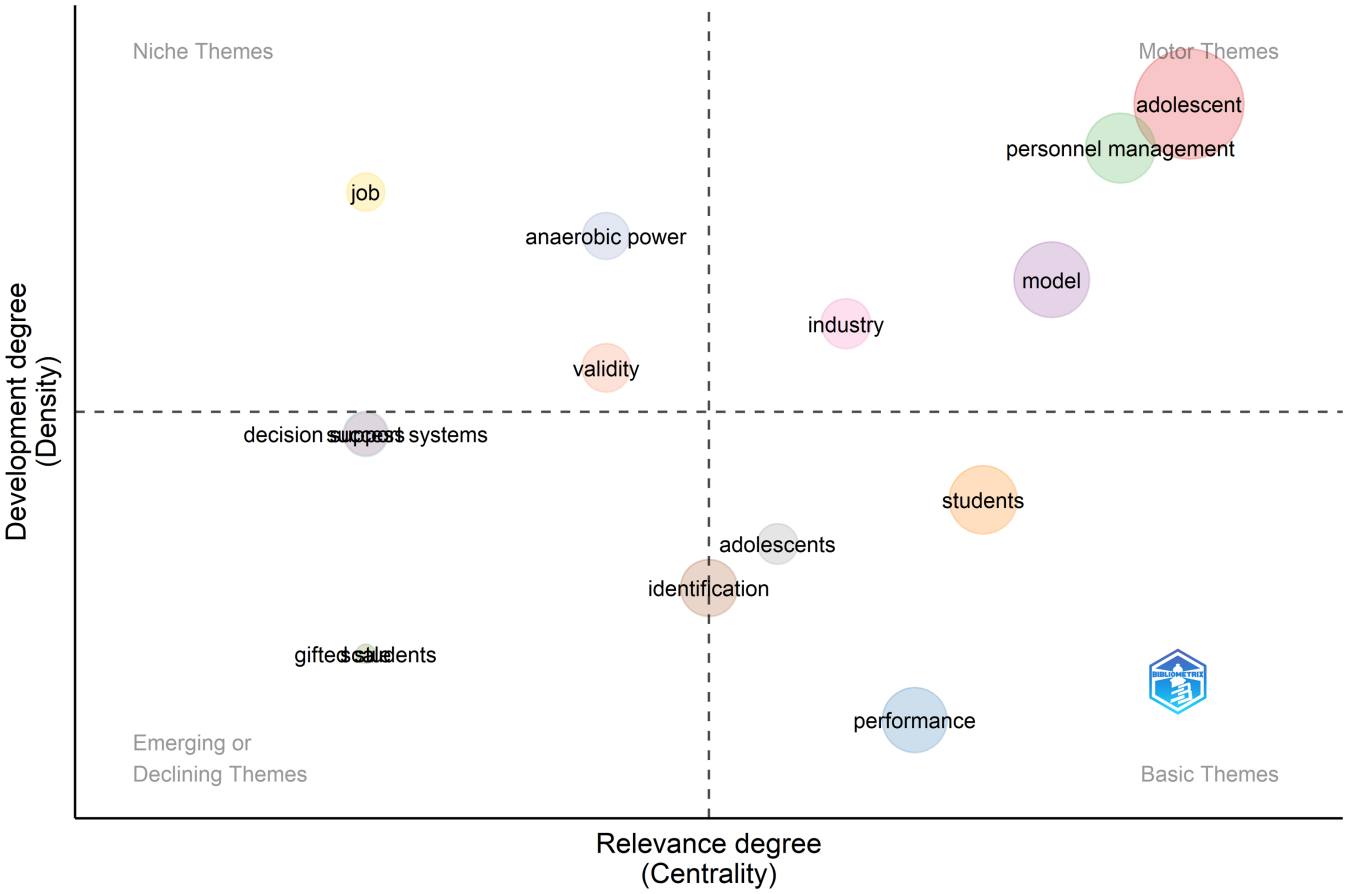
Thematic evolution from 2001 to 2010.

Between 2001 and 2010, researchers’ interest in TI peaked with the publication of 286 articles or 11.4% of all publications (Figure 10). Diversity of topics in all four quadrants showed growing importance and development of TI motor themes which included the study of adolescent characteristics, personnel management, and models of talent identification and selection. Performance became a widely adopted topic in multiple fields of study. Other basic themes included focus on identification programs for students and athletes. Emerging themes included giftedness and competitive advantage. Niche themes included researchers’ specialization in talent at the workplace (job and success), validity of identification measures, and determinants of elite talent such as anaerobic power.

**Figure 11:**
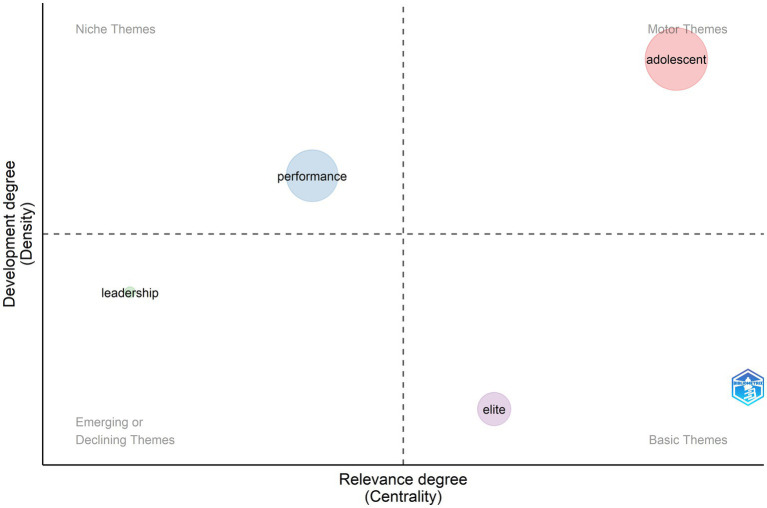
Thematic evolution from 2011 to 2020.

The period between 2011 and 2020 showed great productivity in TI research with 1,559 articles or 62.3% of all publications (Figure 11). The main characteristic of these publications was high specialization within fields. Adolescent development took the forefront as a motor theme with a focus on physiology, sports aptitudes, fitness, early identification, and sports such as soccer. Performance was a growing and topic in the field of management with a focus on work talent, human resources, and talent selection.

**Figure 12:**
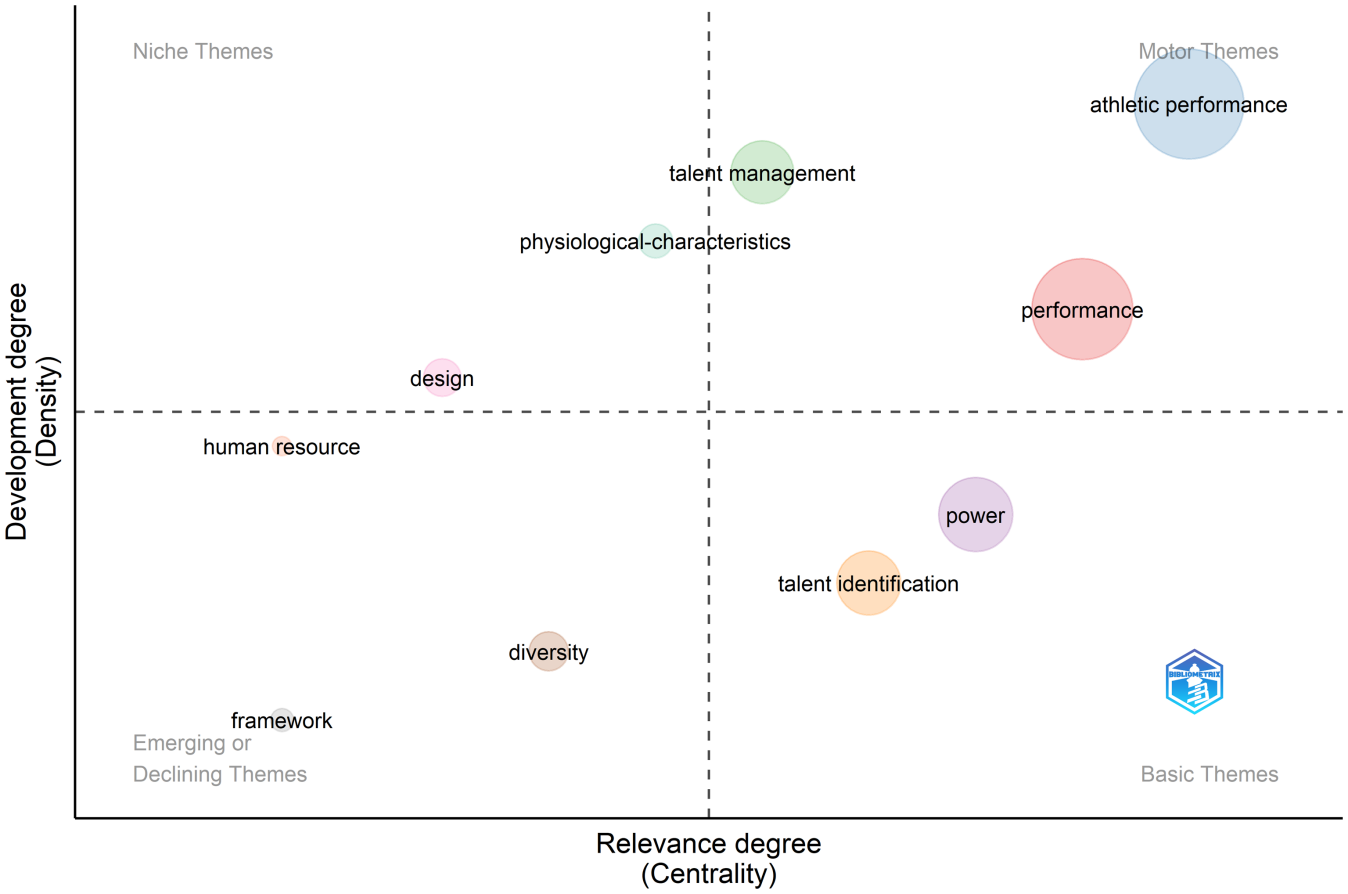
Thematic evolution from 2021 to 2022.

TI research continues its expansion and growth in the last 2 years. Current trends in TI research signal diversification of research topics in niche, motor, basic, and emerging areas (Figure 12). As of November 1, 2022, 595 articles had been published. Strong motor themes like athletic performance in sport sciences and general high performance in management give evidence of the importance of TI for the sport industry and workplace. Talent management has grown to become another motor theme in management and adjacent multidisciplinary fields including keywords like human capital, employment talent, retention, and career development. Talent identification practices (e.g., selection, maturation, talent, validity) and determinants of anaerobic power continue to be widely studied basic themes. Noteworthy emerging and niche themes incorporate issues of diversity (e.g., fairness, representation), frameworks for TI, human resource management, and design of tools and innovations in TI (e.g., identification algorithms, AI, big data).

## 4. Discussion

This bibliometric analysis described trends across 80 years of research in TI represented by over 2,000 articles and more than 5,000 authors. This work contributes to the development of the body of TI research by (a) highlighting the role of TI in multiple disciplines, (b) determining the most impactful sources and authors in TI research, and (c) tracing the evolution of TI and hence identifying gaps and future opportunities in exploring and developing TI research.

### 4.1. The importance of talent identification

TI research and practice continue to play a paramount role for human progress. This fact is evidenced by the increasing adoption of the concept of talent, TI research and practices across the fields of education, psychology, STEM, management, sports, and multidisciplinary areas. A quest for discovering, acquiring, developing, and retaining talent is the cornerstone of the sports and workplace industries (McDonnell, 2011; DeFraine et al., 2014). The often-called “war for talent” is a critical sign that talent is a scarce resource and stakeholders are willing to compete for it (Klett and Wang, 2013). In the fields of management and sports, early identification, training, and retention of talents has situated TI as an essential element of productivity in the workforce (Liu, 2021a; Alanazi, 2022) and elite performance in sports like soccer and basketball (Burgess et al., 2012; Alcântara et al., 2022). Therefore, identifying, selecting, and developing talents has become, more than ever, key to the development of individuals and societies. TI research has transcended fields and reveals that a collective effort among stakeholders, researchers, and practitioners is necessary to detect future elite athletes, innovative scientists, productive as well as competitive workers, and exceptional leaders (Lubinski and Benbow, 2021; Jooss et al., 2022).

### 4.2. Productivity in TI

Our performance analysis of the existing body of TI research, as found in Scopus and WOS, illustrates that although TI originated in psychology and education, it has rapidly permeated two major fields, namely sports science and management. Over 900 journals have included articles about TI, several with high citation indices. For example, the top 30 journals collectively have over 750 publications about TI (*M* = 26, SD = 21). The *Journal of Sport Sciences*, the *International Journal of Human Resource Management*, *Frontiers in Psychology*, and *PloS One* have taken the lead in scientific publications compared to traditional journals devoted to the study of giftedness and high ability like *Gifted Child Quarterly, High Ability Studies*, or *Intelligence*.

While the most productive researchers are nested in management and sports, some of the most co-cited foundational works on talent, TI, and talent development come directly from psychology and education. For example, the work of psychologists and educators has created a bridge for communication across disciplines, for example, Gagne’s work (Gagne, 1997, 2004; Belanger and Gagne, 2006). Widely cited across disciplines, Gagne’s model has influenced TI research through an operational definition of talent and a system of environmental support to nurture talent. The work of Lubinski and colleagues has contributed to understanding the role of cognitive and spatial abilities in the development of expertise in STEM, the workplace, creative pursuits, and the visual arts (Wai et al., 2009; Lubinski, 2010; Kell and Lubinski, 2013). These pivotal contributions have allowed a small number of highly productive researchers in sports and management to create prolific research agendas. Less than ten researchers have trailblazed and established the emerging, basic, and motor themes of TI research. Looking at the top publications, we found longitudinal studies of individuals with great mathematical talent (Lubinski and Benbow, 2006), studies of program effectiveness (Vaeyens et al., 2008; Till and Baker, 2020), optimization of TI processes (Till and Baker, 2020), advocacy for social responsibility in global TI (Bhattacharya et al., 2008), bridges between theory and practice (Abbott and Collins, 2004), or development of integral measures to capture the multidimensional nature of talent (Abbott, 2006; MacNamara and Collins, 2015a).

Although these journals and authors continue to solidify the knowledge structure of TI research, there is room for improvement in the implementation of global TI research and practice. Most bibliometric analyses include country-level article production as a measure of contribution to science. We are sensitive to challenges to scholarly productivity in developing countries and institutions where limited resources and policies influence the number of such contributions. In addition to access, major publication venues are located in industrialized countries which may create barriers to access such as journal database paywalls or language. Moreover, despite the high productivity of researchers, there remained a scarcity of international collaborations. Collaboration efforts occurred more often only among industrialized countries. However, eight countries had only one international collaboration and six did not have collaborations at all. Our analysis describes the advancement and productivity in TI within developed nations, but also raises awareness of the scientific isolation of other countries, which is a common theme among all areas of research. We encourage both cross-country and cross-disciplinary collaborations not only to ensure participation and consensus, but mainly to enhance knowledge production, innovation, and diversity within TI research. Promoting the open production and exchange of knowledge across geographic and disciplinary borders is key to broadly understanding human phenomena, advancing science, and boosting technology within a globalized society (Lin and Li, 2022). As concerns of TI equity and fairness increase, more work is necessary to ensure cross-pollination of ideas and to tackle inequities from a global and multidisciplinary perspective.

### 4.3. Future of TI

As TI research matures, open debates continue to represent challenges to progress in TI. TI researchers’ fixation with age and maturation for identification across the fields of sports, psychology, and education has implications for individual physical and mental health (Matthews and Rhodes, 2020; Bilgiç and Işın, 2022; Towlson et al., 2022). Such challenges call for urgent action on revising the goals of TI according to some authors. A number of articles addressed the goal of early TI as a pathway to excellence, expertise, eminence, or elite performance (Subotnik et al., 2011; Baker et al., 2012; Filipovic et al., 2012; Allen and Hopkins, 2015; Anderson, 2020; Dixson et al., 2020; Abreu, 2021). Through this lens, TI implies the transformation of potential into manifest outcomes in a small, selected group of individuals. However, if such an end goal is not achieved, are the resources allocated to TI wasted? In this regard, critical views of TI have promoted a debate on the necessity of revising TI goals and practices while negotiating stakeholders’ priorities (Pankhurst et al., 2013; Brouwers et al., 2015; McDonnell et al., 2020) and individual well-being (Parry, 2012; Galabova and McKie, 2013; Bjork et al., 2022). To help resolve this debate, Pankhurst and Collins (Pankhurst and Collins, 2013) argue that coherence is needed between research behind TI goals, TI systems and process, and talent development.

Some emerging researchers in sports and management reconceptualize TI as talent management, a perhaps more coherent, eclectic, and humane process (Douglas, 2013). Talent management aligns with identification, development, allocation and relocation of talent that is responsive to contextual needs. This perspective suggests broad TI practices accompanied by diversification in program participation, incorporating the potential to learn, practice, and develop mastery rather than anticipating high performance based on current ability (Christensen, 2009). Moreover, concerns about talent retention in STEM fields may suggest that new generations, for instance Millennial talents, not only look for success and expertise, but collaboration, flexibility, and belonging within a broad conception of deployment of talent and life balance (DeFraine et al., 2014). Bringing more balance into TI and talent development adds flexibility to nurture talent, which may result in the reallocation and retention of acquired talent (Douglas, 2013; DeFraine et al., 2014; Arede et al., 2019; Civera et al., 2020). Ongoing pathways and entry points can reframe TI and subsequent talent development practices as a holistic life-long process in which talent management sits at the intersection of stakeholder needs and healthy individual well-being (MacNamara and Collins, 2015a,b; Haycraft et al., 2019).

Although TI research has evolved in multiple fields, several of TI processes remain obscure and niched within specific fields. To remediate this problem and increase crosspollination of ideas and practices, researchers have called for integration of conceptual foundations across fields. For example, as Faber and colleagues (Faber et al., 2021) suggest in their review of TI in sports and gifted education, consensus building offers opportunities for joint definitions, operationalizations, measurements, and TI programs that foster potential, adding value to our understanding of talent. Similarly, Kalen and collaborators’ (Kalen et al., 2021) meta-analysis synthesizes evidence of the role of cognitive decision-making skills and specific tasks in assessing potential for high performance in sports. Another meta-analysis by Milani and colleagues (Milani et al., 2021) studied the effect of learning agility in developing exceptional leadership. Such findings suggest a need for multidisciplinary research on the validity of constructs related to TI and talent management across organizations and cultures. Because of limited information on cross-field and cross-cultural validity of TI constructs, fostering global multidisciplinary TI research and practices is one possible solution to fostering consensus.

In a globalized society, the identification and acquisition of talent is a priority for organizations and countries (Abeuova and Muratbekova-Touron, 2019). TI and talent development have implications for the integration of human capital into larger initiatives promoting global socioeconomic growth and prosperity (Abeuova and Muratbekova-Touron, 2019). Therefore, much work is needed for understanding the dynamics of TI, talent development and talent management in the context of the migration of exceptional human capital (Shen, 2017; Latukha, 2018; Newton Moses, 2019; Lubinski and Benbow, 2021). The internationalization of talent may also call for the development of policies that encourage transference of talents *via* migration or international cooperation, though competition for talent will continue to be fierce across countries. For instance, the US has historically been a clear beneficiary of brain gain due to immigrant talent in areas such as sports, arts, sciences, leadership, investment, entrepreneurship, technology, and education (West, 2010), though this may shift over time such that other countries may become favored talent hubs. As a result of research, practice, and policy favoring TI, countries and institutions can advance by embracing the idea of global talent.

The disadvantages of human error and limited evidence of validity and reliability call for the development of innovation and technology for TI. Emerging work in the use of sensors and AI (Chua et al., 2017; Marcelino et al., 2020; Fuss et al., 2021) as well as data mining and neural networks (Bush et al., 2015; Lopes et al., 2018; Liu, 2021b) hint at promising advancements in effective TI. Questions about the effectiveness of TI as an individualized practice remind us of the collaborative and dynamic nature of modern societies. Therefore, proposals on collective and group-based TI and talent management could guarantee synergetic systems based on group talent (Mayor Silva, 2015). Qualitative and mixed methods strategies to capture coaches and scouts’ tacit reasoning during talent selection using verbal reports have also shown promise and warrant further exploration to address TI from multiple stakeholder perspectives (Reeves et al., 2019). From ethical and strategic concerns, researchers in the field of management call for early identification of high potential for leadership in the management industry as early as high-school and college (Kotlyar, 2018; Johnson, 2020). Fairness continues to be a priority in TI, as researchers recognize the intersections of talent and disability identification beyond the able-bodied context, including neurodiversity. Basic and motor theme research has begun to focus attention on paralympic competitions through TI and talent development (Dehghansai and Baker, 2020; Dehghansai et al., 2021). Finally, lagging efforts in TI to discover high potential in minoritized populations has led to negative perceptions and misconceptions of TI in the general population in some contexts. Understanding the importance of human progress, old and new researchers will have the challenging task to design mechanisms to promote equity and remove barriers, effectively matching talent with opportunity for development, growth, and exceptional performance (Dawwas, 2022; Farndale et al., 2022; Peterson et al., 2022).

## 5. Limitations

This bibliometric analysis aimed to present an overview of TI research over a period of almost 80 years. To accomplish this goal, we merged two major databases, Scopus and WOS. Carrying out such a comprehensive work has limitations. First, our results and conclusions are restricted in scope to the articles and authors included in these databases. Although these are broad databases, not every article in TI is indexed. While Scopus and WOS are well known, high-quality indexing practices are not a consistent standard in bibliometrics and scientometrics. Items predating 1991 may lack proper meta-data indexation, keywords, DOI, titles, etc. We included articles in multiple languages to consider the widest collection of international items. While this decision was effective to track collaborations and citations across researchers, journals, and countries, bibliometric software is still limited to English-only analysis of keywords and themes. Because the Scopus meta-data does not include information about article or journal field, we extracted field data from the Scimagojr and WOS database. A limitation of this approach is that not all journals in Scimagojr have a clear identification within fields of research. Several journals had multiple classifications across different fields, therefore our classification must be interpreted with caution. Scopus and WOS included all articles containing TI and proxy terms. We eliminated duplicates across the two databases. However, bibliometric analysis cannot distinguish unethical practices such as publishing similar materials in different venues. This is a concern for all bibliometric analyses because the statistical analysis focuses on bibliographic metrics and not the content and quality of the article. Finally, our research focused only on title, abstract and keywords including talent identification and similar terms, such that other literature relevant to talent identification but conceptualized with different terms might have escaped our analysis.

## 6. Conclusion

The first 50 years of TI research drew a slow path to establishing the foundations for subsequent explosive growth and attention from the scientific community. Our findings reveal the top contributing authors, journals, and countries in the collective construction of a global and multidisciplinary TI research community. Network analysis represented co-citation and collaboration networks among researchers signaling influential patterns and trends in TI research. This information can help new and existing researchers in TI identify knowledge funds in the broad multidisciplinary TI literature. The last 30 years have seen dramatic growth and diversification of interests in TI research. TI has a presence in over seven major fields including education, psychology, STEM, sports, management, and multidisciplinary research. Debates about definitions, characteristics, assessment, validity, and diversity in TI continue to be a strong focus of attention for the research community. Crucial to closing gaps, researchers and practitioners may benefit from looking at cross-disciplinary evidence supporting the importance of discovering and nurturing high potential in the pursuit of excellence and human progress. For example, the empirical and conceptual work of researchers in the fields of education and psychology have been pivotal to building bridges across disciplines. These conceptual contributions are key to building consensus and closing gaps through common definitions of potential, talent, performance, and success.

Talent is a multidimensional construct that requires multidimensional and dynamic assessments to be uncovered. Stakeholders can benefit from implementing evidence-based instruments comprising a wider variety of cognitive, physical, and socioemotional abilities to screen and measure the multiple facets of individual talent. In this regard, the fields of sports and management have made important contributions that increase accuracy and predictive power of TI models. For example, TI recruiters can benefit from understanding and adopting new TI technologies. Implementations such as artificial intelligence, complex big data models, and real time performance monitoring offer unique opportunities to understand the evolution of talent and streamline talent selection. As talent development is not a linear and consistent process, researchers and practitioners can create and investigate revolving door systems to nurture individuals’ talent according to their own biological and psychosocial development. TI has consequences that affect individuals beyond the participation in self-contained talent development programs and the geographical boundaries of talent searches. Practitioners and researchers interested in TI and talent development over the lifespan have begun to investigate approaches to adult development, mental health, emerging leadership and eminence. In a globalized world, talent allocation, immigration, and talent wars are themes that will shape human capital transfer through international competition and collaboration. Finally, looking at the future of TI, this study encourages collaboration through cross-pollination of research toward consensus building in concepts, measurement, and execution practices. Our findings indicate that cross-field collaboration can mitigate discipline myopia and scientific isolation of TI researchers. We believe multidisciplinary approaches to TI have the power to yield valid and relevant solutions for the myriad of common problems in TI within currently isolated fields and contexts.

## Data availability statement

The raw data supporting the conclusions of this article will be made available by the authors, without undue reservation.

## Author contributions

FP-M and JW contributed to conception and design of the study. FP-M organized the database, performed analysis, and wrote the first draft of the manuscript. All authors contributed to manuscript revision, read, and approved the submitted version.

## Funding

This research was supported, in part, by a grant (GR016538) from the Virtual Center for Advanced Potential (VCAP) at Schmidt Futures https://www.schmidtfutures.com/our-work/virtual-center-for-advanced-potential/. The funders had no role in study design, data collection and analysis, decision to publish, or preparation of the manuscript.

## Conflict of interest

The authors declare that the research was conducted in the absence of any commercial or financial relationships that could be construed as a potential conflict of interest.

## Publisher’s note

All claims expressed in this article are solely those of the authors and do not necessarily represent those of their affiliated organizations, or those of the publisher, the editors and the reviewers. Any product that may be evaluated in this article, or claim that may be made by its manufacturer, is not guaranteed or endorsed by the publisher.

## Footnotes

^1^ https://rdrr.io/cran/bibliometrix/src/R/metaTagExtraction.R

^2^ https://www.scimagojr.com/journalrank.php
